# Reduced Chlorine in Drinking Water Distribution Systems Impacts Bacterial Biodiversity in Biofilms

**DOI:** 10.3389/fmicb.2018.02520

**Published:** 2018-10-23

**Authors:** Claire Bertelli, Sophie Courtois, Marta Rosikiewicz, Philippe Piriou, Sébastien Aeby, Samuel Robert, Jean-François Loret, Gilbert Greub

**Affiliations:** ^1^Institute of Microbiology, University Hospital Center and University of Lausanne, Lausanne, Switzerland; ^2^Suez CIRSEE, Le Pecq, France

**Keywords:** chlorination, drinking water, biofilm, chlorine, microbiome, 16S rRNA, metagenomics

## Abstract

In drinking water distribution systems (DWDS), a disinfectant residual is usually applied to limit bacterial regrowth. However, delivering water with no or reduced chlorine residual could potentially decrease the selection for antimicrobial resistant microorganisms, favor bacterial regrowth and result in changes in bacterial populations. To evaluate the feasibility of water reduction in local DWDS while ensuring water safety, water quality was measured over 2 months in two different networks, each of them harboring sub-areas with normal and reduced chlorine. Water quality remained good in chlorine reduced samples, with limited development of total flora and absence of coliforms. Furthermore, 16S rRNA amplicon-based metagenomics was used to investigate the diversity and the composition of microbial communities in the sub-networks. Taxonomic classification of sequence reads showed a reduced bacterial diversity in sampling points with higher chlorine residuals. Chlorine disinfection created more homogeneous bacterial population, dominated by *Pseudomonas*, a genus that contains some major opportunistic pathogens such as *P. aeruginosa*. In the absence of chlorine, a larger and unknown biodiversity was unveiled, also highlighted by a decreased rate of taxonomic classification to the genus and species level. Overall, this experiment in a functional DWDS will facilitate the move toward potable water delivery systems without residual disinfectants and will improve water taste for consumers.

## Introduction

Drinking water is one of the most closely monitored resource, strictly regulated by international and national quality standards. Microbial characterization of drinking water mostly relies on conventional culture-based methods, such as heterotrophic plate counts (HPC) and selective plating of coliforms such as *Escherichia coli* (Schets et al., 2002). Avoiding microbial regrowth is important since some bacterial pathogens such as *Legionella pneumophila* and *Mycobacterium avium*, amoeba-infecting bacteria such as *Chlamydia-*related organisms (Greub and Raoult, 2004; Corsaro et al., 2009), or free-living pathogenic amoebae such as *Acanthamoeba, Hartmanella*, and *Naegleria* can grow in drinking water distribution systems (DWDS) and cause waterborne illnesses (Poitelon et al., 2009; Marciano-Cabral et al., 2010; Koubar et al., 2011; Aw and Rose, 2012). A minimal disinfectant residual concentration is usually maintained for this purpose. Excessive microbial growth and accumulation of biofilms can also lead to the deterioration of water quality with associated undesirable visual turbidity, taste, and odors and cause process malfunction such as filter or pipe clogging, biofouling, and enhanced corrosion (Zacheus et al., 2001; Hammes et al., 2008).

Biofilms are structurally complex populations of microorganisms attached to a surface and embedded in a matrix of extracellular polymeric substances (EPS) composed of extracellular DNA, proteins, and polysaccharides (Lopes et al., 2009). The EPS matrix provides physical stability against shear forces and offers a protection to disinfectants (Flemming and Wingender, 2010). The process of biofilm formation on plumbing material in chlorinated DWDS is rapid, reaching 10^7^ cells per cm^2^ within a month (Morvay et al., 2011). An investigation of early stages of biofilm formation in an experimental, chlorinated DWDS showed the occurrence of significant changes in biofilm composition, with a gradual increase in species richness over 28 days (Douterelo et al., 2014). However, stable biofilm formation is a process that can take years (Martiny et al., 2003) owing to the successive colonization by microorganisms slowly providing additional adhesion sites that can be used by further microorganisms (Lee et al., 2008; Andrews et al., 2010). In DWDS, source water parameters (e.g., temperature, pH), as well as pipe characteristics (material, diameter, roughness) and seed bacterial cells can influence the formation of biofilm (Liu et al., 2004; Simões et al., 2007).

The composition of bacterial populations in DWDS is strongly influenced by water quality, and especially by the nature and concentration of disinfectants (Mathieu et al., 2009; Hwang et al., 2012; Pinto V.G. et al., 2012; Vaz-Moreira et al., 2013; Gomez-Alvarez et al., 2015). Within a stable chlorinated DWDS, bacterial composition was shown to remain stable over three seasons (Pinto V.G. et al., 2012). Moreover, the bacterial community in the distribution network reflected that of the drinking water when leaving the treatment plant. More recently, Roeselers et al. (2015) reported the presence of distinct microbial communities in raw and processed water of various non-chlorinated DWDS in the Netherlands. Surprisingly, network-specific communities were stable in time, suggesting that drinking water samples from different distribution systems may be distinguished by microbial profiling.

The absence of pathogen proliferation in unchlorinated distribution systems in The Netherlands indicates that increasing bacterial biodiversity could protect against proliferation of pathogens (Roeselers et al., 2015). This natural protection offered by the presence of colonizing banal bacteria is known as the “protective biofilm” concept, or the “probiotic approach” (Wang et al., 2013). It is corroborated by the observed increase in the proportion of bacteria resistant to antimicrobial agents (chloramphenicol, trimethoprim, and cephalothin) after chlorination (Shi et al., 2013). The use of disinfectants or bacteriostatic agents such as copper also led to a strong reduction in bacterial diversity and a selection of more resistant and pathogenic microorganisms such as *Legionella* and *Mycobacterium* species (Buse et al., 2014). Furthermore, potential plasmids and mobile genetic elements such as insertion sequences were more abundant in chlorinated waters suggesting possible increased spread of antibiotic resistance genes in the microbial population (Shi et al., 2013). Finally, microbial quality of European unchlorinated waters, as evaluated in terms of compliance for fecal indicators, was demonstrated to be equal or superior to that of chlorinated waters (Hambsch et al., 2007). Following the discovery of disinfection byproducts, coupled with negative public perceptions regarding the taste of chlorine, several countries, including the Netherlands, Switzerland, and Germany, have initiated a move toward potable water delivery systems without residual disinfectants. Delivering water with no or reduced chlorine residuals could decrease the selection for antimicrobial resistance in colonizing communities, favor bacterial regrowth, and result in positive changes in bacterial populations.

In this context, this study aimed at investigating whether local facilities could easily deliver safe drinking water with reduced chlorine, hindering potential negative effects of disinfectants. Chlorine concentration was minimized in isolated subnetworks of two DWD S to evaluate its consequences on water quality and microbial communities. 16S rRNA gene amplicon sequencing was used to investigate the diversity and composition of microbial communities in both subnetworks with normal and reduced concentration of chlorine. The results obtained here offer the grounds for a move toward safe potable water delivery systems without residual disinfectants.

## Materials and Methods

### Experimental Area in Drinking Water Distribution Systems

**FIGURE 1:**
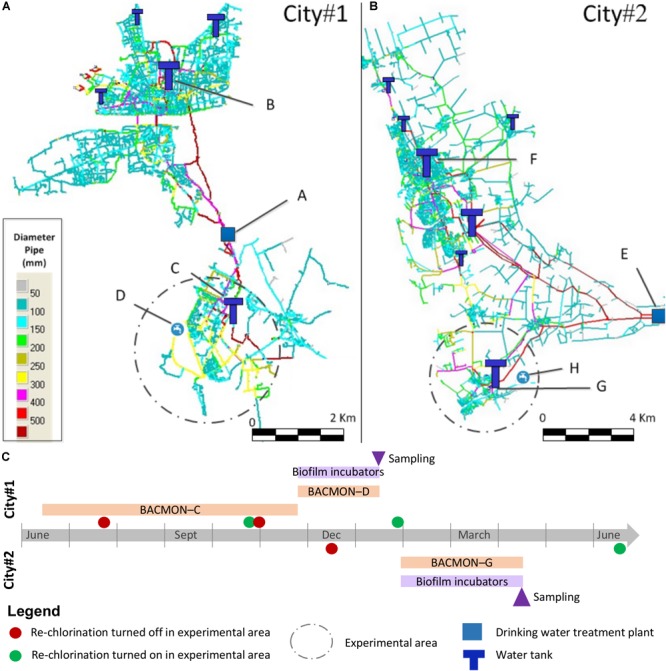
Experimental design and drinking water distribution systems. The drinking water distribution systems (DWDS) of City#1 **(A)** and City#2 **(B)** comprised an experimental zone (dashed circle) where residual chlorine concentration was minimized by turning off the re-chlorination station **(C)**. Four biofilm incubators where added at the outlet of the drinking water treatment plants and in the water network of each city. Biofilms A and E were recovered at the DWTP outlet, B and F downstream a drinking water tank with a rechlorination system, C and G downstream a drinking water tank without chlorine injection, and D and H in the distribution network. Biofilms were recovered after 8 and 10 weeks incubation for City#1 and City#2, respectively. A BACMON online sensor was set-up prior to the experiment in point C, and during the biofilm formation on points D and G.

To evaluate the effect of chlorination on the microbial populations present in DWDS, experimental areas with reduced chlorine concentration were defined in two cities in France and submitted either to normal chlorination or to reduced chlorination (Figure 1). The drinking water treatment plant (DWTP) supplying City#1 uses influenced groundwater as its source and includes the following treatment steps: direct filtration, ozonation, ultrafiltration, and chlorination, with a production capacity of 40,000 m^3^ per day for 155,000 inhabitants. The DWTP supplying City#2 is only composed of groundwater pumping and chlorination, with a mean production of 81,000 m^3^ per day for 220,000 inhabitants. The DWDS are composed of 96% cast iron, 2.5% iron, 1.5% other materials in City#1 and 74% cast iron, 16% polyethylene, 5% iron, 3% asbestos cement, and 2% other materials for City#2.

For each city, a limited experimental area with minimized residual chlorine concentration was defined and isolated from the rest of the network where chlorine residual around 0.2 ppm was maintained (Figure 1, dashed line). Practically, the entry of each zone consists of a drinking water tank (points C and G, respectively) where chlorine injection was turned off. The experimental zone of City#1 represents a network length of 63 km (total network length: 340 km) and served around 19,500 inhabitants while the experimental zone of City#2 represents a network length of 146 km (total network length: 1450 km) and served around 24,000 inhabitants.

### Sample Collection and Physico-Chemical Measurements

Experimental periods with minimized residual chlorine were between July 21st, 2014, and January 27th, 2015, and between December 16th, 2014, and June 30th, 2015, for City#1 and City#2, respectively. After at least 1-month wash out period to allow reduction of free chlorine in the experimental area, eight in-line biofilm incubators using borosilicate glass beads (Loret et al., 2005) were installed for 2 months at each sampling point (for City#1 between November 25th, 2014, and January 19th, 2015, and City#2 between January 29th and April 15th, 2015). Each incubator was composed of a PVC compartment containing 700 autoclaved glass beads of 5 mm diameter (representing a contact surface with the water of 550 cm^2^) and was fed with a constant water flow of 30 L/h during the sampling period. For each sampling point, physico-chemical parameters of water quality such as temperature and free chlorine were measured on grab water samples using a thermocouple thermometer (HI8757 Hanna instruments) and ISO 7393-2, respectively. Periodically, heterotrophic plate counts (HPC) at 22°C and 36°C (ISO 6222:1999) as well as fecal contamination indicators (*E*. *coli* by ISO 9308-1:2000 standard method and enterococci by ISO 7899-2:2000 standard method) were also monitored at the sampling points.

In three biofilm points (C, D, and G), a new on-line bacteria sensor (GRUNDFOS BACMON) was used to measure in a continuous way the concentrations of total bacteria and abiotic particles in bulk water feeding the biofilm incubators. With a 10-min resolution, the system is capable of counting particles in water and classifying them as either bacteria or abiotic particles, based on shape and pattern of light diffraction (Højris et al., 2016). In sampling point C, the system was placed in the period before and during the minimizing chlorine experiment in order to follow the impact of free chlorine concentration on the bacterial biomass.

At the end of the biofilm formation period, the glass beads were collected with 500 mL of drinking water sampled at the same sampling point. After shipping within 24 h in cooling conditions, all the water volume and glass beads were sonicated in water bath (Branson 130 W) for 2 min to recover the biofilm formed at their surface. Three hundred milliliter of this suspension were sent in cooling conditions to the Institute of Microbiology (Lausanne, Switzerland), where samples were stored at 4°C and processed within 3 days.

### Sample Preparation and Sequencing

A 0.22-μm filter was used to filter 150 ml from the water sample with as little non-soluble particle material as possible. After removal, the filter was scraped in 1 ml PBS to resuspend bacteria. DNA was extracted from 500 μl of the previous suspension with the Wizard SV Genomic DNA Purification Sytem (Promega, ref. A2361), protocol for tissue, and eluted in 50 μl TE buffer. The samples were then prepared according to the “16S Metagenomic Sequencing Library Preparation” (Part. # 15044223 Rev. B) from Illumina (San Diego, United States) using the KAPA HiFi HotStart Ready Mix (KAPA Biosystems, United States). Samples were run on gel to verify the amplification of 16S DNA. The PCR used in this study amplifies the region V3 and V4 of the 16S rRNA gene using the best primer pair proposed by Klindworth et al. (2013) and amplifies most known bacterial species: S-D-Bact-0341-b-S-17 CCTACGGGNGGCWGCAG and S-D-Bact-0785-a-A-21 GACTACHVGGGTATCTAATCC. A negative control was performed using the same protocol, starting with the filtering of DNA-free water. No amplification of 16S rRNA was obtained by PCR, and the negative control was thus not processed further. PCR products were purified and prepared for sequencing using 16S Metagenomics Sequencing Library protocol (Illumina, standard protocol). For each city, the four samples were indexed and pooled in a single run of MiSeq 2 × 300 bp for sequencing.

### Bioinformatics and Statistical Analysis

Between 1.6 and 3.5 million reads were obtained for each sample. The quality of raw reads was controlled with FastQC (Andrews, 2010). For each sample, pairs of reads were assembled using PandaSeq (Masella et al., 2012) (parameters: -p CCTACGGGNGGCWGCAG -q GACTACHVGGGTATCTAATCC -A simple_bayesian -l 390 -L 450 -B -N), and the resulting sequences were dereplicated using vsearch (Rognes et al., 2016) (vsearch –derep_fulllength). Sequences for all the samples were then pooled and dereplicated again filtering out singletons (vsearch –derep_fulllength –sizeout –minuniquesize 2), before clustering (vsearch –cluster_size –sizein –id 0.97), removal of chimera (vsearch –uchime_denovo –abskew 2 –sizein), dereplication of nonchimeric reads and relabeling (vsearch –derep_fulllength –sizein –relabel OTU_ –xsize). OTUs were assigned to a taxonomical unit with QIIME (Caporaso et al., 2010) using the RDP classifier (Wang et al., 2007) and the EzBioCloud database (Yoon et al., 2017) for the V3–V4 region. For each sample, the occurrence of OTUs was calculated starting from the dereplicated sequences using the global alignment search of vsearch (Rognes et al., 2016) (vsearch –usearch_global –id 0.97 –strand plus). Results were further processed using R 3.3.3 (Hornik, 2010) with packages dplyr, tidyr, dendextend, ggplot2, RColorBrewer, Vegan, and lazyeval. Distance among samples was calculated using Bray–Curtis model on the presence/absence of each OTU, and the hierarchical clustering was inferred using the Ward.d2 method. Non-metric multidimensional scaling was used to represent differences in microbial composition in two dimensions based on the presence-absence and quantification of OTUs.

A subsampling of read counts to 1 Mio was performed to normalize across samples. The number of different OTUs identified, Shannon or Simpson indexes were not correlated to the number of assembled paired-reads obtained in each sample (correlation -0.04, *p*-value 0.91; 0.56, *p*-value 0.15; and -0.66, 0.07, respectively), before or after rarefaction. Raw read counts for each OTU are available as Supplementary Table S1.

### Sequence Data Availability

Raw sequencing reads were deposited in the European Nucleotide Archive (ENA) under the project number PRJEB27988.

## Results

### Water Quality

**FIGURE 2:**
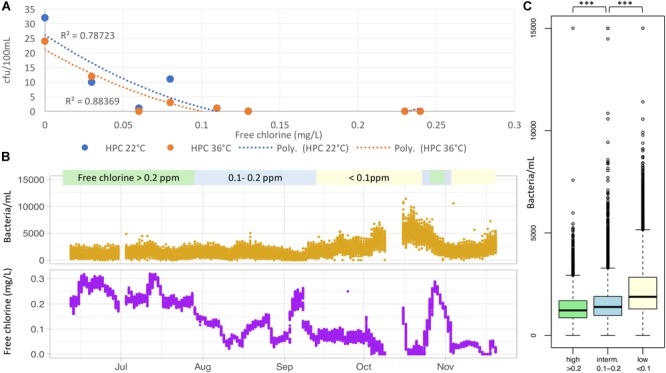
Water quality measures. **(A)** Chlorine concentration is negatively correlated with heterotrophic plate counts (HPC, CFU/100 mL) at different temperatures when considering all samples from both cities. However, HPC remains lower than commonly accepted values at all chlorine concentrations. **(B)** The BACMON online sensor shows the variation in bacteria/ml at the drinking water tank outlet of City#1 (Sample C) following the decrease of chlorine over a 5-month period at the same location. The blank periods in early July and mid-October were due to an accidental switch-off of both on-line sensors. Each sensor generated more than 20.000 measurements. **(C)** Bacterial counts by the BACMON differ significantly between the three categories of chlorine concentration, even without taking into consideration the latency in bacterial regrowth.

The average and maximum values for free chlorine, temperature, and heterotrophic plate counts (HPC) at 22 and 36°C as monitored during the biofilm formation are shown in Table 1. Average temperatures varied from 9°C to 13°C in the DWDS of City#1. The temperature was very constant around 10°C in City#2, if we except sample H located in a public building that reached an average temperature of 18°C. In the main distribution network, the average free chlorine concentration varied between 0.11 and 0.24 mg/L in both cities. In the experimental area with minimized residual chlorine, the average concentration was reduced between 0.06 and 0.08 mg/L in DW Tank outlets, thus representing a three- to fourfold reduction compared to DW Tank outlets in the rest of the network. Furthermore, chlorine concentration was reduced to 0 and 0.03 mg/L in samples further down in the distribution network. As can be expected, HPC at 22°C values are negatively correlated to chlorine concentration (Figure 2A), but remained much lower than the binding limit value 200 cfu/ml at consumer’s tap applied in Denmark where drinking water is based solely on groundwater and distributed without disinfection residual (Miljøstyrelsen, 2017). However, HPC tests also recover microorganisms belonging to the natural and typically non-hazardous microbiota of water. Further analyses of coliforms, including *E. coli* and enterococci, measured in various point of the experimental area during chlorine minimization period did not show any non-conform measures of water quality.

**Table 1:** Water quality parameters during biofilm formation.

City	Sample ID	Biofilm collection date	Location	Minimized residual chlorine	Temperature °C		Free chlorine (mg/L)		HPC 22°C (cfu/100 mL)		HPC 36°C (cfu/100 mL)	
					Mean	SD	Mean	SD	Mean	SD	Mean	SD
City#1	A	January 19th	DWTP outlet	No	13.3	0.6	0.13	0.07	0	0	0	0
	B	January 19th	DW Tank outlet (21,000 m^3^ capacity)	No	11.0	0.1	0.23	0.01	ND	ND	ND	ND
	C	January 19^th^	DW Tank outlet (5,000 m^3^ capacity)	Yes	12.8	0.5	0.06	0.03	1	2	0	0
	D	January 19th	Upstream re-chlorination station	Yes	9.7	1.5	0.00	0.00	32	24	24	19
City#2	E	April 15th	DWTP outlet	No	10.2	0.4	0.11	0.01	1	5	1	3
	F	April 15th	DW Tank outlet (1,500 m^3^ capacity)	No	10.3	0.4	0.24	0.01	0^∗^	0^∗^	0^∗^	0^∗^
	G	April 15th	DW Tank outlet (15,000 m^3^ capacity)	Yes	9.3	0.5	0.08	0.02	11	8	3	3
	H	April 15th	Distribution network	Yes	18.2	5.4	0.03	0.03	10	10	12	10

*ND, not determined. ^∗^Only one value reported during monitoring. Mean and SD values were calculated from measures performed during the biofilm formation experiment.*

As shown in Figure 2B, during the extended experiment period, residual chlorine values at sampling point C varied from 0 to 0.32 mg/L mainly due to a constant dose together with a varying flow at the waterwork. Free chlorine level remained in a relatively stable operation in June and July, before a decrease in August. Responses to changes in the system are slow. Bacterial counts by the BACMON show a steady but slow increase over the 1 month period with low chlorine concentration. However, an increase of chlorine concentration to higher levels (>0.2 mg/L) during only approximately 1 week was sufficient to get the system back at normal operation, with a large decrease in bacterial counts per milliliter. When comparing bacterial concentration in the distribution system at different level of residual chlorine, a statistical difference (*p*-value <2.2e-16) was observed between mean total bacterial counts at low (<0.1 mg/L), intermediate (0.1–0.2 mg/L), and high (>0.2 mg/L) free chlorine concentration (Figure 2C). Real-time monitoring performed under minimized residual chlorine conditions at the two other experimental points (D and G) showed stable bacteria counts along the whole period, excepted punctual variations due to hydraulic events (results not shown).

In addition to these parameters, assimilable organic carbon (AOC) determination was performed on six samples during the experimental period. AOC is usually determined to characterize the ability of water to support bacterial growth. Nitisoravut et al. (1997) proposed a threshold value of 30 μgC/l, considering the analytical sensitivity and variability of AOC determination, to ensure a limited bacteria growth in a non-chlorinated system. AOC was determined according to van der Kooij (1992). The results showed low average AOC concentrations in treated water from DWTP in City #1 and #2, respectively, around 30 and <10 μgC/L.

### Diversity of Microbial Communities

**FIGURE 3:**
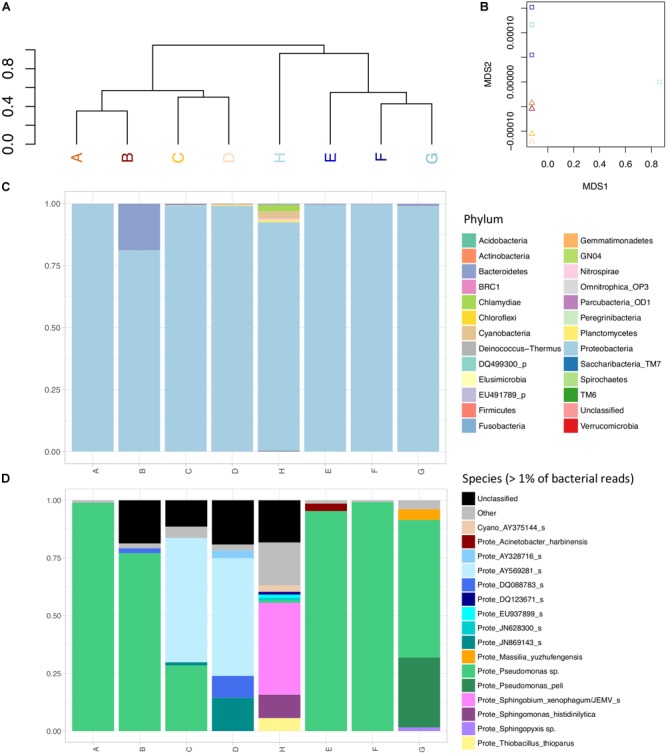
Taxonomic classification of 16S rRNA amplicons. **(A)** Hierarchical clustering of samples based on the presence/absence OTUs for City#1 (brown labels) and City#2 (blue labels) sampling sites. A gradient from dark to light colors reflects chlorine concentration at each site. **(B)** Non-metric multidimensional scaling plot of the sample based on the presence/absence and the quantification of reads classified in bacterial OTUs (Ward distance), displayed with the same color-code as in **(A)**. **(C)** Bacterial composition at the phylum level, showing the large predominance of *Proteobacteria*, and some *Bacteroidetes, Cyanobacteria*, and *Chlamydiales* in a few samples. **(D)** Bacterial composition at the species level, showing the large predominance of *Pseudomonas* spp. in the samples with higher chlorine, and the abundance of unknown proteobacterial clades as well as *Sphingomonales* in samples with lower chlorine concentration. Only identified species encompassing more than 1% of reads were represented in **(D)**.

16S amplicon sequencing was performed to investigate biofilm composition in the DWDS with varying chlorine concentration (Table 2). Whereas sequence assignment to genus level was successful for over 95% of the dataset, species-level assignment was more challenging, due to the presence of sequences with lower than 97% identity to known bacterial species or sequences equally distant to known bacterial species. Using hierarchical clustering on the presence or absence of OTUs, we observed two major groups corresponding to the two cities (Figure 3A), indicating that each city has a signature composition of bacteria in its DWDS. This signature could also be affected by the sampling at different time of the year (Dec–Jan and Feb–April). Samples with similar concentrations of chlorine formed secondary groups in City#1 only. The large number of bacterial phyla identified in the samples suggests this hidden diversity of lowly abundant species that contribute to forming the city signature (Figure 3C).

**Table 2:** Sequencing results of samples with reduced (^+^) or normal chlorine concentration.

Sample ID	Raw reads after QC	Assembled reads	Genus^∗^	Species^∗^	No OTUs°	Shannon index	Simpson index
**A**	3,456,830	2,697,877	100.00	99.87	67	0.086	0.022
**B**	2,549,074	2,002,047	100.00	80.85	75	0.739	0.371
**C**^+^	2,417,863	1,857,971	99.99	87.85	125	1.386	0.623
**D**^+^	1,606,927	1,170,238	100.00	80.05	104	1.639	0.692
**E**	2,929,766	2,558,840	99.98	99.61	174	0.903	0.522
**F**	3,331,979	2,905,145	99.98	99.75	93	0.833	0.399
**G**^+^	1,886,251	1,577,627	99.97	98.88	134	1.691	0.758
**H**^+^	2,311,235	2,030,530	95.04	75.28	1038	2.772	0.807

***^+^**Reduced chlorine concentration. ^∗^Reported as the percentage of reads classified at the taxonomic level among those belonging to kingdom “Bacteria.” °After rarefaction.*

A similar clustering taking into account the frequency of each OTU, rather grouped samples with high chlorine (A, F, B, E, and to a lesser extent G) and those with lower chlorine (C, D, H), notably reflecting the large predominance of *Pseudomonas* spp. in the samples with high chlorine (Figure 3D). The non-metric multidimensional scaling highlights the particular bacterial communities found in sample H (Figure 3B). Sample H from the distribution network of City#2 exhibits a large number of OTUs present in very small proportion in the sample forming a biofilm with very particular bacterial composition that could be linked to the sampling point (end of distribution network).

Samples with low chlorine harbored significantly higher bacterial diversity calculated with Shannon (*p*-value = 0.009502) or Simpson (*p*-value = 0.01435) index that account for both abundance and evenness of OTUs in the samples (Table 2). Pearson’s correlation coefficient between chlorine concentration and Shannon index was -0.65 but did not reach statistical significance due to the low number of samples considered here (*p*-value = 0.07987). A larger sample size would have been required to increase the power of the study and thus the significance of the observed correlation and differences. The higher bacterial diversity in samples with lower chlorine concentration comprised several clades of poorly described proteobacteria from the order *Sphingomonadales.* The many known and unknown species recovered in the various samples were particularly prevalent in those with lower chlorine concentration, although they were also present in small proportion in other samples.

### *Chlamydia, Legionella, Mycobacterium*, and *Pseudomonas* in the Microbial Communities

A closer analysis of bacterial phyla identified in these water samples showed the presence of several known colonizers and potential pathogens of water environments (Table 3). Obligate intracellular bacteria belonging to the order *Chlamydiales* were observed in six samples mostly in very small amounts only retrieved due to the high number of reads per sample. However, sample H showed a large diversity of OTUs classified as belonging to the *Chlamydiales* order. Members of the family *Parachlamydiaceae* were the most common (35 OTUs), followed by *Rhabdochlamydiaceae* (17 OTUs). One OTU of an unknown *Parachlamydiaceae* was particularly prevalent with over 2% of the sample H reads. These so-called *Chlamydia-*related bacteria are known to grow steadily within amoebae, and in particular *Acanthamoeba* spp., that are found ubiquitely in water environments (Greub and Raoult, 2004; Kebbi-Beghdadi and Greub, 2014). The pathogenic potential of some of these organisms is still debated, but many strains are proposed to be rather environmental isolates with no, or little, pathogenic potential (Horn, 2008; Greub, 2009; Taylor-Brown et al., 2015). Known pathogens such as *Legionella pneumophila* or *Legionella lytica* were only observed as traces once (0.001% of reads), although other known and unknown *Legionella* species most likely also growing in amoebae were recovered at very low concentration (up to 0.3% of reads) in five samples. Their occurrence in potable water plumbing systems has been widely reported, and legionella are known to thrive in warmer and stagnant water (Loret and Greub, 2010; Jjemba et al., 2015). The higher temperature observed in sampling point H, as well as its particular situation in the DWDS could explain the higher prevalence and diversity of legionella and chlamydia observed (Table 3). Unknown *Mycobacterium* spp. were recovered in six different samples comprising only up to 0.1% of the sample reads. Finally, reads classified as *Pseudomonas aeruginosa* were present in all samples of City#1 and sample G of City#2 in very small proportion (between 0.0001 and 0.01%).

**Table 3:** Number of distinct OTUs (>97% identity) for major water pathogens and chlamydia-related bacteria.

Order	Family, genus, or species	A	B	C	D	E	F	G	H
*Actinomycetales*	*Mycobacterium*		1	2	1	1		1	2
*Chlamydiales*	***Criblamydiaceae***								2
	***Parachlamydiaceae***			2	2	3			35
	*Metachlamydia*								1
	*Neochlamydia*					1			1
	*Parachlamydia*			1					2
	*Protochlamydia*								10
	*Rhabdochlamydiaceae*								17
	*Simkaniaceae*								1
	*Waddliaceae*	1					1		
*Legionellales*	*Legionellaceae*			4	2	3		2	38
*Pseudomonadales*	*Pseudomonas aeruginosa*	1	1	1	1			1	

*Families are highlighted in bold, whereas genus and species are written with normal case.*

## Discussion

The presence of bacteria in drinking water networks has been recorded in numerous systems throughout the world, whether residual disinfectants are applied or not. Bacteria disinfectant and water age were both observed to be strong factors in shaping bacteria and eukaryotic community structures (Wang et al., 2014; Gomez-Alvarez et al., 2016). We have investigated here the changes in bacterial concentration after reduction of chlorine concentration in subnetworks of DWDS, as well as the composition of biofilms after a 2-month incubation in several points of the DWDS serving about 20,000 inhabitants in two cities. The results discussed below are coherent with previous observations, although statistical significance of 16S based observations was limited by the low number of samples collected in this study.

Real-time monitoring performed under minimized residual chlorine showed stable bacteria counts along the whole period at points D and G. In one of the sampling points, the results showed an increase in total bacterial cell counts with an online BACMON system as well as HPCs at different temperatures. However, values remained within the limits of water quality, such as the 200 cfu/ml used as a cutoff in Denmark where no chlorination of water is performed. Moreover, microorganisms recovered through HPC tests generally include those that are part of the natural (typically non-hazardous) microbiota of water. Evidence supports the conclusion that, in the absence of fecal contamination, there is no direct relationship between HPC values in ingested water and human health effects in the population (Bartram et al., 2003).

Opportunistic pathogens that may be recovered among HPC microbiota include strains of *P. aeruginosa, Acinetobacter* spp., *Aeromonas* spp., *Klebsiella pneumoniae, Legionella*, etc. In this study, no *Klebsiella* or *Aeromonas* spp. were identified using the 16S amplicon sequencing. A few *Acinetobacter harbinensis* usually found in water samples (Li et al., 2014) as well as another unknown species have been identified. Traces of *P. aeruginosa* as well as *Legionella* spp. in the range of 10^-2^–10^-5^ percent of the reads were observed in a few samples and likely do not represent any risk for public health. Similar abundance of *Legionella* spp. around 0.003% were previously reported in unchlorinated water (Liu et al., 2017). A higher diversity and up to 0.3% of the reads for un unknown species of *Legionella* was identified in sample H that was located at the end of the DWDS and harbored particularly high temperature. This sample hence harbored a very different microbial community that could be rather influenced by its physico-chemical properties and water flow than by the chlorination level in this experiment.

In unchlorinated DW systems (Liu et al., 2017), bacterial diversity in biofilms was characterized with similar numbers of OTUs (446 to 1416 OTUs) as in the present study. We observed lower diversity of bacterial communities in samples with high chlorine concentrations compared to those in the experimental zone without re-chlorination. Similar results were obtained, although with even greater diversity (Shannon index between 2.45 and 3.16) in the study of Gomez-Alvarez et al. (2016) describing the response of microbial communities in bulk water and biofilm phase to changes of operational parameter in chloraminated systems. They observed that biofilms sampled immediately after a chlorine burn were composed of low diverse communities of closely related taxa, while bulk water were represented with highly diverse populations of phylogenetically over-dispersed communities.

Bacteria are present in the bulk water but also within sediments and in the form of biofilms attached to the inside of distribution pipe walls. Most frequent bacteria groups in (bulk) drinking waters are Gram-negative bacteria members of the phylum *Proteobacteria*, mainly of the classes alpha, beta, and gamma (Hoefel et al., 2005; Pinto A.J. et al., 2012; Vaz-Moreira et al., 2013), as also observed in this study. Mathieu et al. (2009) reported that alphaproteobacteria were more sensitive to residual chlorine (0.4 mg/L) than beta and gammaproteobacteria. *Pseudomonas* resistance to free chlorine is associated to their capacity to form biofilm. Suboptimal chlorine treatment of drinking water was shown to lead to the selection of multidrug-resistant *Pseudomonas aeruginosa* (Shrivastava et al., 2004). All chlorine-resistant isolated strains produced mucoid colonies characterized by an overproduction of extracellular polysaccharide alginate (Grobe et al., 2001). Alginate-containing slime confers protection on *P. aeruginosa* against chlorine and may contribute to survival of these bacteria in chlorinated water systems (Shrivastava et al., 2004). Pronounced capsule layer (as observed on swimming pool isolates of *P. aeruginosa*) may also provide protection against disinfectants or antiseptics (Seyfried and Fraser, 1980).

Similar to the situation observed in sample B, *Bacteroidetes* also dominated microbial communities together with proteobacteria in a recent study (Liu et al., 2017). The presence of large proportions of *Hyphomicrobium* and *Sphingomonadaceae* were previously suggested to be likely due to the biofilm inoculum originating from chlorinated water that selects for bacterial species able to survive by acclimating to the residual levels or traveling through the system protected by particles (Williams et al., 2005). Indeed, members of the *Sphingomonadaceae* are known to be resistant to chlorine and were suggested to be a reservoir of antimicrobial resistance genes (Vaz-Moreira et al., 2011). Communities observed in the present study as well as previous studies investigating the effect of changes in chlorination level only represent a snapshot of bacterial biofilm formation in a short time frame. The long-term development of more stable and diversified bacterial communities, and its potential convergence toward microbial communities observed in countries where DWDS are free of chlorine are to be investigated further as other parameters such as water origin, flow, or pipe material can lead to major change heavily influence microbial communities. Shifts in proteobacterial composition within the biofilm were reversible when exposed to discontinuous chlorination (Mathieu et al., 2009). The resilience of microbial communities to frequent changes in oxidant stress questions the emergence of populations more resistance to chlorine, another argument in favor of a stable decrease, and perhaps abolishment of chlorine in DWDS. Finally, under certain conditions biofilms harboring mixed population may limit the survival of enteric bacterial pathogens that could be introduced in DWDS by intrusion for example (Banning et al., 2003), providing a desired protective effect.

## Conclusion

The reduction of chlorine in two functional local DWDS facilities enabled us to evaluate the influence on bacterial quantity and communities while ensuring water quality and safety, with the absence of coliforms. 16S amplicon sequencing enabled us to document a decreased bacterial diversity in highly chlorinated samples, that were dominated by *Pseudomonas* spp. The reduction of chlorine in potable water delivery systems is a desirable objective that could contribute to the decrease of antimicrobial resistance genes in the microbial communities suggested by other studies (Shi et al., 2013; Jia et al., 2015) and enhance the development of a diversified protective biofilm (Wang et al., 2013). Moreover, it would enhance consumer experience whose main complaint is often the taste of chlorine in tap water.

## Author Contributions

PP designed and SR performed the sampling. SA processed the samples and performed the sequencing. MR and CB developed the bioinformatic pipeline for data analysis. CB and SC performed the data analysis and drafted the manuscript. GG and J-FL designed, funded, and supervised the project. All authors corrected and approved the final manuscript.

## Conflict of Interest Statement

The authors declare that the research was conducted in the absence of any commercial or financial relationships that could be construed as a potential conflict of interest.

## Supplementary Material

TABLE S1 OTU counts and taxonomic assignment for all samples.

## References

[B1] Andrews J. S. Rolfe S. A. Huang W. E. Scholes J. D. Banwart S. A. (2010). Biofilm formation in environmental bacteria is influenced by different macromolecules depending on genus and species. *Environ. Microbiol.* 12 2496–2507. 10.1111/j.1462-2920.2010.02223.x 20406292

[B2] Andrews S. (2010). *FastQC: A Quality Control Tool for High Throughput Sequence Data. Babraham Bioinforma 1.* Available at: http://scholar.google.com/scholar?hl=en&btnG=Search&q=intitle:FastQC+a+quality+control+tool+for+high+throughput+sequence+data.#0

[B3] Aw T. G. Rose J. B. (2012). Detection of pathogens in water: from phylochips to qPCR to pyrosequencing. *Curr. Opin. Biotechnol.* 23 422–430. 10.1016/j.copbio.2011.11.016 22153035 PMC7126744

[B4] Banning N. Toze S. Mee B. J. (2003). Persistence of biofilm-associated *Escherichia coli* and *Pseudomonas aeruginosa* in groundwater and treated effluent in a laboratory model system. *Microbiology* 149 47–55. 10.1099/mic.0.259380 12576579

[B5] Bartram J. Cotruvo J. Exner M. Fricker C. Glasmacher A. (2003). *Heterotrophic Plate Counts and Drinking-water Safety | IWA Publishing.* Available at: https://www.iwapublishing.com/books/9781843390251/heterotrophic-plate-counts-and-drinking-water-safety [Accessed July 25 2018]. 10.1016/j.ijfoodmicro.2003.08.005 15145582

[B6] Buse H. Y. Lu J. Lu X. Mou X. Ashbolt N. J. (2014). Microbial diversities (16S and 18S rRNA gene pyrosequencing) and environmental pathogens within drinking water biofilms grown on the common premise plumbing materials unplasticized polyvinylchloride and copper. *FEMS Microbiol. Ecol.* 88 280–295. 10.1111/1574-6941.12294 24490699

[B7] Caporaso J. G. Kuczynski J. Stombaugh J. Bittinger K. Bushman F. D. Costello E. K. (2010). QIIME allows analysis of high-throughput community sequencing data. *Nat. Methods* 7 335–336. 10.1038/nmeth.f.303 20383131 PMC3156573

[B8] Corsaro D. Feroldi V. Saucedo G. Ribas F. Loret J.-F. Greub G. (2009). Novel Chlamydiales strains isolated from a water treatment plant. *Environ. Microbiol.* 11 188–200. 10.1111/j.1462-2920.2008.01752.x 18793313

[B9] Douterelo I. Sharpe R. Boxall J. (2014). Bacterial community dynamics during the early stages of biofilm formation in a chlorinated experimental drinking water distribution system: implications for drinking water discolouration. *J. Appl. Microbiol.* 117 286–301. 10.1111/jam.12516 24712449 PMC4282425

[B10] Flemming H.-C. Wingender J. (2010). The biofilm matrix. *Nat. Rev. Microbiol.* 8 623–633. 10.1038/nrmicro2415 20676145

[B11] Gomez-Alvarez V. Humrighouse B. W. Revetta R. P. Santo Domingo J. W. (2015). Bacterial composition in a metropolitan drinking water distribution system utilizing different source waters. *J. Water Health* 13 140–151. 10.2166/wh.2014.057 25719474

[B12] Gomez-Alvarez V. Pfaller S. Pressman J. G. Wahman D. G. Revetta R. P. (2016). Resilience of microbial communities in a simulated drinking water distribution system subjected to disturbances: role of conditionally rare taxa and potential implications for antibiotic-resistant bacteria. *Environ. Sci. Water Res. Technol.* 2 645–657. 10.1039/C6EW00053C

[B13] Greub G. (2009). Parachlamydia acanthamoebae, an emerging agent of pneumonia. *Clin. Microbiol. Infect.* 15 18–28. 10.1111/j.1469-0691.2008.02633.x 19220336

[B14] Greub G. Raoult D. (2004). Microorganisms resistant to free-living amoebae. *Clin. Microbiol. Rev.* 17 413–433. 10.1128/CMR.17.2.413-433.2004 15084508 PMC387402

[B15] Grobe S. Wingender J. Flemming H.-C. (2001). Capability of mucoid *Pseudomonas aeruginosa* to survive in chlorinated water. *Int. J. Hyg. Environ. Health* 204 139–142. 10.1078/1438-4639-00085 11759157

[B16] Hambsch B. Böckle K. van Lieverloo J. H. M. (2007). Incidence of faecal contaminations in chlorinated and non-chlorinated distribution systems of neighbouring European countries. *J. Water Health* 5(Suppl. 1) 119–130. 10.2166/wh.2007.143 17890841

[B17] Hammes F. Berney M. Wang Y. Vital M. Köster O. Egli T. (2008). Flow-cytometric total bacterial cell counts as a descriptive microbiological parameter for drinking water treatment processes. *Water Res.* 42 269–277. 10.1016/j.watres.2007.07.009 17659762

[B18] Hoefel D. Monis P. T. Grooby W. L. Andrews S. Saint C. P. (2005). Profiling bacterial survival through a water treatment process and subsequent distribution system. *J. Appl. Microbiol.* 99 175–186. 10.1111/j.1365-2672.2005.02573.x 15960678

[B19] Højris B. Christensen S. C. B. Albrechtsen H.-J. Smith C. Dahlqvist M. (2016). A novel, optical, on-line bacteria sensor for monitoring drinking water quality. *Sci. Rep.* 6:23935. 10.1038/srep23935 27040142 PMC4819223

[B20] Horn M. (2008). Chlamydiae as symbionts in eukaryotes. *Annu. Rev. Microbiol.* 62 113–131. 10.1146/annurev.micro.62.081307.162818 18473699

[B21] Hornik K. (2010). *The Comprehensive R Archive Network.* New York, NY: John Wiley & Sons.

[B22] Hwang C. Ling F. Andersen G. L. LeChevallier M. W. Liu W.-T. (2012). Microbial community dynamics of an urban drinking water distribution system subjected to phases of chloramination and chlorination treatments. *Appl. Environ. Microbiol.* 78 7856–7865. 10.1128/AEM.01892-12 22941076 PMC3485970

[B23] Jia S. Shi P. Hu Q. Li B. Zhang T. Zhang X.-X. (2015). Bacterial community shift drives antibiotic resistance promotion during drinking water chlorination. *Environ. Sci. Technol.* 49 12271–12279. 10.1021/acs.est.5b03521 26397118

[B24] Jjemba P. Johnson W. Bukhari Z. LeChevallier M. (2015). Occurrence and control of *Legionella* in recycled water systems. *Pathogens* 4 470–502. 10.3390/pathogens4030470 26140674 PMC4584268

[B25] Kebbi-Beghdadi C. Greub G. (2014). Importance of amoebae as a tool to isolate amoeba-resisting microorganisms and for their ecology and evolution: the Chlamydia paradigm. *Environ. Microbiol. Rep.* 6 309–324. 10.1111/1758-2229.12155 24992529

[B26] Klindworth A. Pruesse E. Schweer T. Peplies J. Quast C. Horn M. (2013). Evaluation of general 16S ribosomal RNA gene PCR primers for classical and next-generation sequencing-based diversity studies. *Nucleic Acids Res.* 41:e1. 10.1093/nar/gks808 22933715 PMC3592464

[B27] Koubar M. Rodier M.-H. Garduño R. A. Frère J. (2011). Passage through *Tetrahymena tropicalis* enhances the resistance to stress and the infectivity of *Legionella pneumophila*. *FEMS Microbiol. Lett.* 325 10–15. 10.1111/j.1574-6968.2011.02402.x 22092856

[B28] Lee J.-W. Nam J.-H. Kim Y.-H. Lee K.-H. Lee D.-H. (2008). Bacterial communities in the initial stage of marine biofilm formation on artificial surfaces. *J. Microbiol.* 46 174–182. 10.1007/s12275-008-0032-3 18545967

[B29] Li W. Zhang D. Huang X. Qin W. (2014). *Acinetobacter harbinensis* sp. nov., isolated from river water. *Int. J. Syst. Evol. Microbiol.* 64 1507–1513. 10.1099/ijs.0.055251-0 24478215

[B30] Liu G. Tao Y. Zhang Y. Lut M. Knibbe W.-J. van der Wielen P. (2017). Hotspots for selected metal elements and microbes accumulation and the corresponding water quality deterioration potential in an unchlorinated drinking water distribution system. *Water Res.* 124 435–445. 10.1016/J.WATRES.2017.08.002 28787681

[B31] Liu Y.-Q. Liu Y. Tay J.-H. (2004). The effects of extracellular polymeric substances on the formation and stability of biogranules. *Appl. Microbiol. Biotechnol.* 65 143–148. 10.1007/s00253-004-1657-8 15197510

[B32] Lopes F. A. Morin P. Oliveira R. Melo L. F. (2009). Impact of biofilms in simulated drinking water and urban heat supply systems. *Int. J. Environ. Eng.* 1 276–294. 10.1504/IJEE.2009.027805

[B33] Loret J.-F. Greub G. (2010). Free-living amoebae: biological by-passes in water treatment. *Int. J. Hyg. Environ. Health* 213 167–175. 10.1016/J.IJHEH.2010.03.004 20418158

[B34] Loret J. F. Robert S. Thomas V. Cooper A. J. McCoy W. F. Lévi Y. (2005). Comparison of disinfectants for biofilm, protozoa and *Legionella* control. *J. Water Health* 3 423–433. 10.2166/wh.2005.047 16459847

[B35] Marciano-Cabral F. Jamerson M. Kaneshiro E. S. (2010). Free-living amoebae, *Legionella* and *Mycobacterium* in tap water supplied by a municipal drinking water utility in the USA. *J. Water Health* 8 71–82. 10.2166/wh.2009.129 20009249

[B36] Martiny A. C. Jørgensen T. M. Albrechtsen H.-J. Arvin E. Molin S. (2003). Long-term succession of structure and diversity of a biofilm formed in a model drinking water distribution system. *Appl. Environ. Microbiol.* 69 6899–6907. 10.1128/AEM.69.11.6899-6907.2003 14602654 PMC262284

[B37] Masella A. P. Bartram A. K. Truszkowski J. M. Brown D. G. Neufeld J. D. (2012). PANDAseq: paired-end assembler for illumina sequences. *BMC Bioinformatics* 13:31. 10.1186/1471-2105-13-31 22333067 PMC3471323

[B38] Mathieu L. Bouteleux C. Fass S. Angel E. Block J. C. (2009). Reversible shift in the α-, β- and γ-proteobacteria populations of drinking water biofilms during discontinuous chlorination. *Water Res.* 43 3375–3386. 10.1016/j.watres.2009.05.005 19539973

[B39] Miljøstyrelsen (2017). *The Danish Act on Drinking Water Quality and Surveillance: “Bekendtgørelse om Vandkvalitet og Tilsyn med Vandforsyningsanlæg.” Miljø- og Fødevaremin., Miljøstyrelsen, j.nr. SVANA-400-00054 (in Danish) BEK nr. 11.* Available at: https://www.retsinformation.dk/Forms/R0710.aspx?id=194227

[B40] Morvay A. A. Decun M. Scurtu M. Sala C. Morar A. Sarandan M. (2011). Biofilm formation on materials commonly used in household drinking water systems. *Water Sci. Technol. Water Supply* 11 252. 10.2166/ws.2011.053 17223387

[B41] Nitisoravut S. Wu J. S. Reasoner D. J. Chao A. C. (1997). Columnar biological treatability of AOC under oligotrophic conditions. *J. Environ. Eng.* 123 290–296. 10.1061/(ASCE)0733-93721997123:3(290)

[B42] Pinto A. J. Xi C. Raskin L. (2012). Bacterial community structure in the drinking water microbiome is governed by filtration processes. *Environ. Sci. Technol.* 46 8851–8859. 10.1021/es302042t 22793041

[B43] Pinto V. G. Heller L. Bastos R. K. X. (2012). Drinking water standards in South American countries: convergences and divergences. *J. Water Health* 10 295–310. 10.2166/wh.2012.087 22717755

[B44] Poitelon J.-B. Joyeux M. Welté B. Duguet J.-P. Peplies J. DuBow M. S. (2009). Identification and phylogeny of eukaryotic 18S rDNA phylotypes detected in chlorinated finished drinking water samples from three Parisian surface water treatment plants. *Lett. Appl. Microbiol.* 49 589–595. 10.1111/j.1472-765X.2009.02710.x 19793192

[B45] Roeselers G. Coolen J. van der Wielen P. W. J. J. Jaspers M. C. Atsma A. de Graaf B. (2015). Microbial biogeography of drinking water: patterns in phylogenetic diversity across space and time. *Environ. Microbiol.* 17 2505–2514. 10.1111/1462-2920.12739 25581482

[B46] Rognes T. Flouri T. Nichols B. Quince C. Mahé F. (2016). VSEARCH: a versatile open source tool for metagenomics. *PeerJ* 4:e2584. 10.7287/PEERJ.PREPRINTS.2409V1 27781170 PMC5075697

[B47] Schets F. M. Nobel P. J. Strating S. Mooijman K. A. Engels G. B. Brouwer A. (2002). EU drinking water directive reference methods for enumeration of total coliforms and *Escherichia coli* compared with alternative methods. *Lett. Appl. Microbiol.* 34 227–231. 10.1046/j.1472-765x.2002.01075.x 11874547

[B48] Seyfried P. L. Fraser D. J. (1980). Persistence of *Pseudomonas aeruginosa* in chlorinated swimming pools. *Can. J. Microbiol.* 26 350–355. 10.1139/m80-057 6773651

[B49] Shi P. Jia S. Zhang X.-X. Zhang T. Cheng S. Li A. (2013). Metagenomic insights into chlorination effects on microbial antibiotic resistance in drinking water. *Water Res.* 47 111–120. 10.1016/j.watres.2012.09.046 23084468

[B50] Shrivastava R. Upreti R. K. Jain S. R. Prasad K. N. Seth P. K. Chaturvedi U. C. (2004). Suboptimal chlorine treatment of drinking water leads to selection of multidrug-resistant *Pseudomonas aeruginosa*. *Ecotoxicol. Environ. Saf.* 58 277–283. 10.1016/S0147-6513(03)00107-6 15157584

[B51] Simões L. C. Simões M. Oliveira R. Vieira M. J. (2007). Potential of the adhesion of bacteria isolated from drinking water to materials. *J. Basic Microbiol.* 47 174–183. 10.1002/jobm.200610224 17440920

[B52] Taylor-Brown A. Vaughan L. Greub G. Timms P. Polkinghorne A. (2015). Twenty years of research into Chlamydia-like organisms: a revolution in our understanding of the biology and pathogenicity of members of the phylum Chlamydiae. *Pathog. Dis.* 73 1–15. 10.1093/femspd/ftu009 25854000

[B53] van der Kooij D. (1992). Assimilable organic carbon as an indicator of bacterial regrowth. *J. Am. Water Works Assoc.* 84 57–65. 10.2307/41293634

[B54] Vaz-Moreira I. Egas C. Nunes O. C. Manaia C. M. (2013). Bacterial diversity from the source to the tap: a comparative study based on 16S rRNA gene-DGGE and culture-dependent methods. *FEMS Microbiol. Ecol.* 83 361–374. 10.1111/1574-6941.12002 22938591

[B55] Vaz-Moreira I. Nunes O. C. Manaia C. M. (2011). Diversity and antibiotic resistance patterns of Sphingomonadaceae isolates from drinking water. *Appl. Environ. Microbiol.* 77 5697–5706. 10.1128/AEM.00579-11 21705522 PMC3165245

[B56] Wang H. Edwards M. A. Falkinham J. O. Pruden A. (2013). Probiotic approach to pathogen control in premise plumbing systems? *Rev. Environ. Sci. Technol.* 47 10117–10128. 10.1021/es402455r 23962186

[B57] Wang H. Masters S. Edwards M. A. Falkinham J. O. Pruden A. (2014). Effect of disinfectant, water age, and pipe materials on bacterial and eukaryotic community structure in drinking water biofilm. *Environ. Sci. Technol.* 48 1426–1435. 10.1021/es402636u 24401122

[B58] Wang Q. Garrity G. M. Tiedje J. M. Cole J. R. (2007). Naive Bayesian classifier for rapid assignment of rRNA sequences into the new bacterial taxonomy. *Appl. Environ. Microbiol.* 73 5261–5267. 10.1128/AEM.00062-07 17586664 PMC1950982

[B59] Williams M. M. Santo Domingo J. W. Meckes M. C. (2005). Population diversity in model potable water biofilms receiving chlorine or chloramine residual. *Biofouling* 21 279–288. 10.1080/08927010500452695 16522541

[B60] Yoon S.-H. Ha S.-M. Kwon S. Lim J. Kim Y. Seo H. (2017). Introducing EzBioCloud: a taxonomically united database of 16S rRNA gene sequences and whole-genome assemblies. *Int. J. Syst. Evol. Microbiol.* 67 1613–1617. 10.1099/ijsem.0.001755 28005526 PMC5563544

[B61] Zacheus O. M. Lehtola M. J. Korhonen L. K. Martikainen P. J. (2001). Soft deposits, the key site for microbial growth in drinking water distribution networks. *Water Res.* 35 1757–1765. 10.1016/S0043-1354(00)00431-0 11329678

